# Weight Stigma in the Development, Maintenance, and Treatment of Eating Disorders: A Case Series Informing Implications for Research and Practice

**DOI:** 10.1007/s10802-024-01260-3

**Published:** 2024-11-01

**Authors:** Rachel Kramer, Catherine R. Drury, Sarah Forsberg, Lindsey D. Bruett, Erin E. Reilly, Sasha Gorrell, Simar Singh, Lisa Hail, Kimberly Yu, Rachel M. Radin, Jessica Keyser, Daniel Le Grange, Erin C. Accurso, Kathryn M. Huryk

**Affiliations:** Department of Psychiatry and Behavioral Sciences, University of California, San Francisco, Nancy Friend Pritzker Building, 675 18th St., San Francisco, CA 94143 USA

**Keywords:** Weight stigma, Eating disorders, Adolescence, Treatment

## Abstract

Weight-centric health practices are based on the principle that excess weight predicts chronic disease, informing a growing sociopolitical movement to address an “obesity epidemic.” This hyper-focus on preventing obesity may contribute to weight stigma (i.e., the devaluation and discrimination of individuals based on body size) and other iatrogenic outcomes for youth, including the development and maintenance of eating disorders (EDs). Current evidence-based treatments for EDs include language and practices that may reinforce fears of fatness, body shame, and unhealthy dietary restriction without guidance on addressing weight stigma. Here, we present case examples from three adolescent patients across ED presentations and body sizes to (1) elucidate the role of weight stigma in ED development, (2) highlight the ubiquity and harms of weight stigma within ED treatments, and (3) outline thoughtful protocol adaptations to avoid further harm and facilitate recovery. We conclude with a call for immediate action to advance research characterizing the harms of weight-centric approaches in existing ED interventions to reduce the risk of iatrogenic effects on youth with EDs and advance weight-inclusive approaches to ED treatment.

**Supplementary Information:**

The online version contains supplementary material available at 10.1007/s10802-024-01260-3.

## Introduction

The “obesity epidemic” and the related focus on higher body weight as a danger to health has become a pervasive narrative in medicine, social media, and society as a whole (Bristow et al., 2020; Hardy, 2022; Kornfield et al., 2015; Tomiyama et al., 2013). Indeed, the United States has devoted significant funding and research attention to the promotion of weight loss and obesity prevention (defined as a body mass index [BMI] ≥ 30 kg/m^2^ [(Centers for Disease Control (CDC), 2022)] across healthcare practices and government-supported initiatives, particularly for youth (CDC, 2011; Flegal et al., 2013; Hampl et al., 2023; Temple, 2023). BMI, a crude marker of obesity, is a primary metric of these efforts and the most frequently used criterion for distinguishing between “healthy” and “unhealthy” weight (Gutin, 2018). Despite its prominence, BMI is now recognized to be a poor indicator of individual health (Tomiyama et al., 2016). It does not account for individual variability (e.g., in age, gender, body composition) and other factors (e.g., weight cycling, health behaviors, cardiometabolic health) that are more likely to influence health (Gutin, 2018; Heymsfield et al., 2016; Peterson & Savoie Roskos, 2023; Tylka et al., 2014).

Weight-centric health practices are also ineffective for sustained BMI reduction and can negatively impact general well-being and metabolic health (Bacon & Aphramor, 2011; McEntee et al., 2023; Nuttall, 2015). Specifically, weight loss interventions demonstrate limited short- and long-term efficacy, contribute to weight cycling, and usually lead to a net weight gain versus loss (Brownell & Rodin, 1994; Tomiyama et al., 2013). In addition to limited efficacy, weight-centric approaches contribute to weight stigma (i.e., devaluating and discriminating individuals based on body weight and shape; Brewis et al., 2011) and internalized weight bias (i.e., applying negative stereotypes and attitudes about weight to oneself; Bristow et al., 2020; Durso & Latner, 2008) which are linked to physical (Pearl & Puhl, 2018; Puhl & Suh, 2015) and psychological harm (Brochu, 2020; Emmer et al., 2020; Magson & Rapee, 2022; Tomiyama et al., 2018). Weight stigma in adolescence is associated with suicidality, anxiety, depression, and avoidance of medical care (Brochu, 2020; Magson & Rapee, 2022; Sonneville et al., 2024; Wetzel & Himmelstein, 2023) and predicts chronic disease risk (over and above the risk associated with obesity status), likely through stress-mediated pathways (McEntee et al., 2023; Puhl & Heuer, 2009; Tomiyama et al., 2018). Additionally, weight-based discrimination may shorten life expectancy, even more so than other forms of discrimination and similar to other established risk factors (disease burden, smoking) with a 60% increased risk of mortality after accounting for BMI (Sutin et al., 2015).

## Impact of Weight Stigma on ED Behaviors and Cognitions

Well-intentioned weight loss efforts in youth may inadvertently encourage ED behaviors and cognitions (Golden et al., 2016; Leme et al., 2020) and are a known predictor of ED development (Stice, 2002). Experiences of weight stigma and internalized weight bias, which often precipitate attempts to lose weight, are further associated with body dissatisfaction and ED behaviors that increase ED risk, particularly for individuals in larger bodies (Cerolini et al., 2024; Sonneville et al., 2024). Weight bias in healthcare settings (Lawrence et al., 2021) and inadequate training in EDs (Kazdin et al., 2017) may further hinder providers’ capacity to detect disordered eating, particularly among individuals in larger bodies (Silbiger, 2024), resulting in longer illness durations, delayed referral to ED treatment, and greater body mass loss at presentation to care (Lebow et al., 2015; Moskowitz & Weiselberg, 2017). Notably, the negative impact of weight bias on clinical care extends beyond EDs, as providers who are overly focused on weight or BMI may overlook the true causes of medical symptoms or underlying disease (Phelan et al., 2015). Some providers may even dismiss ED behaviors as “normative discontent” with one’s body in the context of pervasive diet culture.

## Atypical Anorexia Nervosa: An Eating Disorder Mired in Weight Stigma

The diagnostic system for EDs assigns a diagnosis of “atypical anorexia nervosa” to those who present at a higher weight with all of the other symptoms of anorexia nervosa (AN), even though atypical AN is more common (Harrop et al., 2021) and associated with commensurate symptoms severity, medical complications, and mental health sequelae (Garber et al., 2019; Golden & Walsh, 2024; Kramer et al., 2023). This weight-based distinction can reinforce the notion that individuals with atypical AN are not as “ill,” thereby compounding problems with detection and access to care (Sim et al., 2023).

Treatment studies that include youth with atypical AN are nascent (Harrop, 2019; Harrop et al., 2021; Strand et al., 2020). As such, there is no consensus on how to identify treatment goal weight or define weight restoration among youth with atypical AN or higher weight growth trajectories (Jhe et al., 2022) as most existing studies largely focused on AN, have used weight-based population norms (e.g., median BMI^1^; Le Grange et al., 2019). Additionally, studies lack long-term follow up (see Hughes et al., 2017; Lebow et al., 2019) and still report weight outcomes using weight relative to age norms (e.g., mBMI or BMI percentile), rather than more individualized measurements. Such factors contribute to uncertainty about defining weight restoration in this population (Strand et al., 2020).

## Weight Stigma in ED Treatment

Current clinical guidelines for mental health treatment emphasize the importance of attending to minority stress and diversity concerns (Clauss-Ehlers et al., 2019); however, evidence-based treatments for EDs do not directly target body diversity and the experience of weight stigma and bias in their approach. Indeed, manualized ED treatments such as Family-Based Treatment (FBT; Lock & Grange, 2015) and Enhanced Cognitive Behavioral Therapy (CBT-E; Dalle Grave & Calugi, 2020; Fairburn, 2008) do not provide clinicians with adequate guidance on how to address body diversity and current or historical experiences of weight stigma as they relate to ED symptoms, treatment progress, and recovery. Preliminary clinical recommendations for addressing weight stigma during ED treatment have emerged (Dimitropoulos et al., 2019; Kimber et al., 2019; Kramer, 2023) and include recommendations that clinicians provide families with psychoeducation correcting for weight stigma and ensuring all care providers share consistent messaging with families about the need for weight gain to historical growth trajectories. Yet these recommendations do not address all concerns that can arise related to weight stigma. Thus, clinicians must decide when to deviate from treatment protocols to avoid iatrogenic effects, and how to respond to weight stigma within case formulation and treatment.

Additionally, some ED interventions include language and practices that may harm youth by perpetuating weight stigma. For example, evidence-based ED treatments do not typically stipulate an inclusive assessment of historical body weight and may make categorical assumptions about body size. In CBT-E (Dalle Grave & Calugi, 2020), it is recommended that “underweight” patients aim to restore weight to the 25th BMI-for-age percentile, with the rationale that this is sufficient for most cases and that higher weight targets are unrealistic. This approach does not account for adolescents whose historical weight trajectory exceeds this threshold and restoring weight only to the 25th percentile may place them at medical and psychological risk (Seetharaman et al., 2017). Moreover, the manual’s guidance surrounding weight restoration overemphasizes arbitrary BMI thresholds (Flegal et al., 2013) and neglects a more nuanced discussion of weight-based treatment goals (Jhe et al., 2022; Loeb et al., 2023).

When addressing body image concerns, CBT-E interventions do not account for historic body size and assume that aspects of ED pathology such as “feeling fat” (a construct predominantly studied in individuals in smaller bodies; Anderson et al., 2022), engaging in body comparisons, or body avoidance are cognitive distortions of actual body size (Dalle Grave & Calugi, 2020). Thus, patients are typically encouraged to challenge these misperceptions through behavioral experiments and strategies to identify and disprove fears underlying these symptoms. Although some intervention targeting these aspects of negative body image is warranted, current strategies fail to address the experienced and internalized weight stigma that may contribute to these fears and do not consider how “feeling fat” may be experienced differently by an individual in a larger body (Anderson et al., 2022), and may inadvertently reinforce societal beliefs that being at a higher weight is something to be feared.

Potential for iatrogenic harm related to these oversights may be even more likely given weight stigma exists across mental health *and* medical professions. Several studies illustrate high levels of explicit and implicit weight bias among medical doctors and dieticians (Lawrence et al., 2021); weight bias also influences the attitudes and decision-making of mental health professionals (Davis-Coelho et al., 2000) and ED providers (Puhl et al., 2014; Silbiger, 2024), which may contribute to inappropriate care and poor patient outcomes.

## Case Studies

We present three anonymized case examples to provide context on how weight stigma contributes to ED onset and creates challenges in treatment. In each case, treatment adaptations and thoughtful collaboration with an interdisciplinary treatment team were necessary to mitigate harm and facilitate ED recovery. Cases included adolescents and caregivers presenting to care at an Adolescent Medicine clinic in an urban academic medical center with integrated mental health treatment provided by ED-specialized psychologists. In addition to routine medical and therapy visits, patients and families met with a dietitian and psychiatrist on an as-needed basis. For each case, we discuss weight stigma and how it impacted individuals prior to starting ED therapy (i.e., History) and their treatment and recovery (i.e., Treatment). An overall summary (i.e., Summary) follows. Table 1 provides an overview of the impact of weight stigma throughout care (e.g. etiology, diagnosis and assessment, medical care, family and peer response, clinical challenges, modifications to address weight stigma during treatment, and recovery) for each case example. Supplement 1 provides more information on the evidence-based ED treatments utilized across cases. After reviewing our study proposal, the Institutional Review Board determined the study was “not human subjects research” given the project was focused on quality improvement and program development. However, to protect the confidentiality of patients and families, we did not include patient age, race, or ethnicity in the descriptions, and we altered family structure and BMI-based information to similarly classify folks based on CDC weight categories. While we encourage looking at individual features of ED severity (e.g. behaviors, weight loss instead of %mBMI, and weight suppression), we included mBMI/BMI percentile for several reasons. Including mBMI/BMI percentile provides context for how individual norms (growth trajectory) may vary from population norms (e.g. mBMI). Further, mBMI and BMI percentile are still frequently used in research and clinical practice, and we lack a consensus on best practices for how to measure ED severity to account for weight stigma in the field. Ultimately, all patients needed to return to their historic growth trajectories for recovery.

## Case 1

“Megan” was living with her family and was referred by her pediatrician for malnutrition, having lost 21.25% of her body weight in one year, and presented to our clinic at the 49.6th BMI-for-age percentile (21 kg/m^2^; 99.8% median BMI). Megan reported dietary restriction and daily self-induced vomiting episodes and laxative misuse, along with chewing and spitting and once-monthly objective binge episodes. Like many individuals with ED, Megan endorsed a fear of weight gain and persistent difficulty recognizing the seriousness of her illness. She also experienced overvaluation of body weight/shape that contributed to significant functional impairment, and may have been heighted because her body diverged from the idealized thin physique. Megan’s psychologist diagnosed her with atypical AN binge/purge subtype, major depressive disorder (MDD), and posttraumatic stress disorder (PTSD).

### History

Megan had minimal health concerns growing up and reportedly enjoyed eating a variety of foods. Throughout childhood, her BMI-for-age percentile tracked between the 50–85th percentiles; in early adolescence, she gained weight, peaking at 93rd BMI percentile at the peak of her puberty. During this time, she disliked her appearance and experienced weight-based bullying, and started to believe people did not want to be her friend because of her body size. Megan internalized beliefs that she was “too big” and needed to lose weight. She began skipping meals and counting calories, and losing some weight. A few years later, Megan told her pediatrician about her body image concerns and weight-based bullying at school but did not screen positive for an ED when administered a standardized primary care screening tool (Morgan et al., 2000).^2^ The doctor assured Megan her weight (70th BMI-for-age percentile) was “healthy” and encouraged regular, balanced eating. Her physician recognized symptoms of an ED the following year when she returned with continued weight loss and markedly worsened depression. Megan was subsequently hospitalized for suicidal behavior and ultimately referred to residential ED treatment, where she restored weight to the 80th percentile and worked to reduce ED behaviors. Megan was discharged prematurely from residential treatment due to lack of continued insurance coverage as is common for individuals in larger bodies (Harrop et al., 2023). After completing a virtual day-program, she entered outpatient ED therapy in the clinic.

### Treatment

Outpatient FBT (Supplement 1) was used to help Megan eat regularly and abstain from binge/purge symptoms. Open weights (i.e., seeing the weight) in session, typical practice in FBT, was very distressing for Megan. Megan’s mother tried to reduce Megan’s distress by providing reassurance that she would help her daughter “get back in shape” by exercising more and “eating healthy.” As Megan’s weight increased beyond her estimated treatment goal weight range, Megan experienced shame and guilt, believing that she had done something wrong. She eventually disclosed that she had been binge-eating more frequently but was too ashamed to tell anyone because she “felt fat.” Subsequently, the therapist and family made a shared decision to temporarily discontinue open weights with careful consideration of the risks and benefits.

After Megan made progress with eating independently in FBT, treatment transitioned to CBT-E (Supplement 1) to address body dissatisfaction and urges to engage in ED behaviors when unsupervised. Megan began using CBT-E skills to cope with ED thoughts and urges, and her weight stabilized at the 92nd BMI-for-age percentile. She reported shame and guilt for eating due to others’ negative comments about her eating, experienced different treatment because of her weight, and reported ongoing urges to engage in ED behaviors to be thinner and better liked.

Although not an intervention explicitly described in the CBT-E manual, the therapist provided psychoeducation on body diversity and the impact of experiences of weight stigma. This included teaching Megan that human bodies come in a wide range of shapes and sizes, which are all worthy of kindness and respect. The therapist helped Megan engage in critical thinking about the relationship between weight and health, as well as learning how to identify and challenge automatic negative thoughts related to body size that were reflective of implicit weight stigma. The therapist also supported Megan in recognizing that weight stigma is a form of discrimination wherein the target is often held personally responsible for their mistreatment, rather than placing this onus on society to change. These concepts were incorporated into the shared case conceptualization of Megan’s ED, and she identified how internalized weight stigma contributed to her ED behaviors. Eventually, Megan could tolerate open weights and challenge her fear that regular eating would result in exponential weight gain, and her binge eating reduced. She learned to contextualize critical comments about her body and eating as manifestations of weight stigma, rather than internalizing that something about her was wrong, and attempted to practice body neutrality (i.e., a focus on appreciating and respecting the body without positive or negative judgments; Pellizzer & Wade, 2023). This shift in mindset helped Megan re-engage in sports with peers. ED treatment concluded approximately one year after her initial diagnosis, her weight had stabilized at the 95th BMI-for-age percentile (29.6 kg/m^2^; 141% median BMI) which tracked along her historic growth trajectory, and she transitioned to treatment for depression and PTSD. Just before terminating, she shared with her ED provider that a new pediatrician had advised her to lose weight and “eat healthier.” A chart review revealed that she continued to receive feedback about being “obese” and recommendations to lose weight, regardless of the purpose of her visit. She also indicated continued body image distress and negative self-judgments about “being fat.”

### Summary

Experienced and internalized weight stigma directly contributed to the onset and maintenance of Megan’s ED and negatively impacted her illness course. The weight stigma embedded in ED screening and diagnosis contributed to delayed identification and referral. Because Megan did not access ED treatment until she was experiencing acute psychiatric instability, she required intensive treatment which likely disrupted normal adolescent social development. Intensive care was cut short due to insurance limitations that were likely weight-driven (Harrop et al., 2023). Her therapist made necessary modifications to evidence-based outpatient treatment to meet her needs and mitigate harm as a young woman recovering from an ED in a larger body. Despite her therapist’s ongoing consultation with caregivers and allied health providers about the importance of regular eating and weight stability for maintaining her recovery, Megan continued to receive messages from trusted adults and peers that she needed to lose weight, ultimately increasing her vulnerability to relapse.

## Case 2

“Emily” was a middle school student who was parent-referred to treatment for selective eating. She presented at the 13th BMI-for-age percentile (16.9 kg/m^2^; 86.3% median BMI) and was medically stable, with vital signs and laboratory work within normal ranges. Emily’s dietary repertoire included eight specific food items, limited to starch, dairy, or fruit. She had never tolerated vegetables or most proteins, including meat. She denied weight/shape concerns and other ED behaviors. Emily was diagnosed with avoidant/restrictive food intake disorder (ARFID), with no co-occurring mental health diagnoses.

### History

Emily had a lifelong history of “picky” eating, likely due to sensory sensitivities and low appetite, which her parents and doctors expected her to outgrow. Her BMI continuously tracked at the low end of the growth curve. As she aged, Emily tolerated small portions of an even smaller range of foods, and she would go long periods without eating if her preferred foods were unavailable. Her parents would restrict her access to preferred foods, as they believed them to be fundamentally “unhealthy” (e.g., tortilla chips, milkshakes). Emily was an avid athlete, engaging in 1–2 hours of rigorous activity daily. Emily’s parents learned about ARFID at an educational event around the same time that Emily began experiencing social impairment related to her selective eating. They consulted an endocrinologist who expressed concern for bone health and pubertal development, recommending increased caloric intake. Emily later met with an outpatient dietician, with whom she worked with for seven months before being referred to a specialty clinic.

### Treatment

The specialty clinic recommended CBT for ARFID (CBT-AR***,*** Thomas & Eddy, 2018; Supplement 1) to help Emily increase overall food intake, broaden dietary variety, and support coping with social eating. Although medical providers recommended increasing caloric intake to support growth toward the 15th BMI-for-age percentile, Emily’s parents disagreed and asserted that she did not need to gain any weight. Consistent with the CBT-AR manual, treatment first focused on developing a pattern of regular eating with preferred foods. When Emily increased her caloric intake, her weight and height increased, and she began menstruating, suggesting health benefits associated with increased nutrition. However, both Emily and her parents expressed anxiety about her continued weight gain. Emily shared that her family’s values included being “good, healthy people,” which she explained necessitated being “thin and fit” and only eating “healthy foods.”

Throughout treatment, Emily and her parents had difficulty thinking flexibly about how to define “healthy eating” in the context of a developing teenage athlete with ARFID. This was particularly challenging when parents were asked to obtain foods that Emily could eat at school, as she would only tolerate pre-packaged snacks in that setting. They also questioned the health value of nutritional supplement shakes that were advised to support Emily's high energy needs. To address this, Emily’s clinician helped her family recognize that applying strict dietary rules for the purposes of “health” limited Emily’s ability to eat enough to gain weight, improve her health, and function in school. Her clinician also shared psychoeducation which challenged societal attributions that linked morality to healthy eating.

In later stages of treatment, Emily worked on expanding food variety through in-session and at-home exposures. Despite a strong disgust response to novel foods, she incorporated new vegetables, fruits, and starches into her diet, and new sources of protein and fat (e.g., nuts, cheeses). Emily learned to tolerate variations to preferred foods, which helped to improve her ability to eat available foods in social situations. Treatment concluded when she felt she had made sufficient progress. She discharged at the 36th BMI-for-age percentile (BMI 17.4 kg/m^2^; 95.15% median BMI).

### Summary

Although Emily did not directly experience weight stigma herself, little action was taken to address her chronically poor nourishment and subsequent low weight due to societal messaging about her low weight being “healthy” and an implicit belief that greater morality is tied to thinness (Ringel & Ditto, 2019). Her parents did not recognize her weight as unhealthy and openly disagreed with her treatment team that weight gain should be a treatment goal. Despite Emily’s low weight, these beliefs perpetuate weight stigma and internalized weight bias by implying that any amount of weight gain, regardless of one’s weight, is undesirable. Emily subsequently experienced anxiety about transgressing her family’s values and becoming “unhealthy” after gaining minimal weight in treatment. Efforts to improve food intake and establish patterns of regular eating were further hindered by familial beliefs about which foods were considered “healthy.” Because evidence-based treatment manuals for ARFID do not directly address different definitions of “health,” providers relied on clinical judgment to help the family navigate these challenges. Some ARFID protocols reference the U.S. Department of Agriculture’s “MyPlate” guidelines (Rarback, 2013) which were developed to prevent childhood obesity. This messaging may reinforce an over-focus on balanced nutrition, which may increase youth and caregiver anxiety about an appropriate and realistic diet in the context of expected food flexibility in ARFID recovery.

## Case 3

“Eva” was first assessed and diagnosed with AN, restricting type upon admission to a medical stabilization unit, presenting with bradycardia and hypotension secondary to a 35-pound weight loss. She lived with her family at home and was in middle school at the time.

### History

As a child, Eva’s family members often commented on her weight and she experienced teasing by peers. Throughout her childhood, she tracked along the 89th BMI-for-age percentile; at inpatient admission, she had fallen to the 20th BMI-for-age percentile (17.4 kg/m^2^, 95.5% of median BMI). She described wanting to look more like her peers, who she noted were from different racial/ethnic backgrounds and “tiny.” She noted that family members commented on her “big appetite” and compared her to her siblings, whom she described as thinner. She endorsed skipping lunch and avoiding foods she deemed unhealthy. At the time of her hospitalization, she described one experience of binge eating, no self-induced vomiting, and a pattern of compulsive exercise.

### Treatment

Following hospital discharge, Eva was referred for FBT and participated with her family. Eva’s parents shared that in their family culture, it was normative to comment about weight, and comments were not meant to be critical but considered terms of endearment. Eva's parents struggled to understand her level of distress, expressing the belief that her temperament made her “too sensitive.” In FBT, Eva made excellent progress with early weight gain while simultaneously articulating her fears about eating specific types of food and subsequent changes to her weight/shape. As a result, an exposure-based framework was used first to reduce her anxiety around fear foods. As Eva was able to eat a variety, she was given more autonomy with meals, and her therapist started to focus on body image-related cues triggering distress during therapy. Eva’s mother supported Eva in these exposures, such as shopping for new clothing. In response to Eva’s continued fears of weight gain***,*** the therapist coached Eva’s mother to shift her response from reassurance (e.g., “Don't worry about weight gain, you are beautiful as you are”) to reduce stigmatizing language and provide validation for the underlying causes of Eva’s fears (i.e., experiences of weight stigma by loved ones and peers, leading to feelings of shame about her body shape and size).

Toward the end of treatment, Eva reported significant improvement in cognitive symptoms and was not engaging in ED behaviors; however, despite some weight gain, she did not return to her prior growth curve, ending treatment at the 55th BMI percentile. Given she was medically stable and meeting all other markers of recovery from AN, her medical team did not emphasize the need for continued weight restoration. The treating psychologist highlighted the importance of continued weight gain, consistent with normative adolescent development, noting that Eva may benefit from returning to her prior growth curve to further solidify recovery. Although Eva experienced ongoing anxiety around weight gain, the behaviors maintaining these fears (e.g., avoidance of feared foods, body checking, restrictive eating) were successfully targeted, and the family decided to discontinue therapy.

Approximately 1.5 years after completing FBT, Eva’s mother reconnected to treatment due to a relapse in Eva's restrictive eating and new onset of binge eating behaviors. Eva had maintained her weight around the 50th BMI percentile for a year prior to returning to in-person school following the COVID-19 pandemic. However, her eating routine was disrupted upon returning to school, leading her to unintentionally miss meals and snacks. She began snacking more in the afternoons and evenings, initially not worrying about these behaviors until she noticed weight gain and “panicked.” Eva subsequently started to skip meals and snacks and began an intense 1.5-hour  daily exercise routine, which triggered a cycle of regular binge episodes and increased efforts to restrict eating.

At intake, Eva presented at the 86th BMI percentile. She expressed motivation to stop binge eating, recognizing that restrictive eating was contributing to feeling out of control when she did eat. CBT-E was initiated with parental support to address binge eating disorder (BED) and co-occurring symptoms of anxiety and depression. A shared case conceptualization of her ED was developed, revealing weight-stigma, and uncovering additional information about traumatic events contributing to feelings of worthlessness and body mistrust. As such, her CBT-E case conceptualization was adapted to explore the impact of trauma, cultural identities, and experiences of minority stress which contributed to internalized stigma and disordered eating. The clinician worked from a trauma-informed lens, helping Eva to develop a sense of safety in her environment, while working with trusted adults to address patterns of invalidation of emotion and minimizing the impact of traumatic stressors. The therapist provided education to the family on the role of weight stigma in perpetuating appearance ideals and beliefs about health. Eva’s mother was encouraged to support Eva in eating restricted foods to combat her belief that limiting these foods would help alleviate Eva’s distress and reduce the likelihood of binge eating. As opposed to initial environmental support to restrict access to binge foods, Eva was encouraged to communicate with her mother about ways she could support her to interrupt binge episodes to reduce feelings of shame associated with prior criticism of her eating habits. Eva continuously expressed a desire to avoid body image triggers (e.g., knowing her weight). Pros and cons of open weighing were discussed throughout treatment, and Eva continued to maintain that blind weights were supportive of her recovery. She was open to some body image exposures (e.g., wearing certain clothes, going to social events once avoided due to body shame). Eva determined that other exposures related to developing a non-judgmental stance towards her body were not helpful to her recovery at the time. Thus, treatment focused on developing self-compassion to reduce internalized weight stigma and increase self-worth and self-kindness. Eva and her mother were introduced to principles of weight neutrality (Bacon & Aphramor, 2011), and other educational resources on the historical origins and harms of the thin ideal. Eva completed related cognitive dissonance exercises, including listing 10 things others can do to resist the thin ideal, and developing counter verbal responses to personal experiences of weight stigma.

### Summary

This case illustrates the limitations of evidence-based ED treatments in addressing experiences of weight stigma in various contexts, as well as the risk for relapse and diagnostic migration when these symptoms remain unaddressed. Although Eva met the standard benchmarks for recovery upon completion of FBT, she remained weight suppressed (Singh et al., 2021), thus increasing her risk for ED maintenance (Lowe et al., 2018). At second presentation, modifications to the treatment plan were made to mitigate the repetition of harms associated with weight stigma since weight stigma likely played a role her relapse, such that while she made significant progress in FBT, she and her family, along with her treatment team, did not feel it was important to prioritize further weight gain. While this decision was in part driven by her progress in all other areas of recovery, Eva and her parents were reluctant to pursue further weight gain given her prior experiences of weight-based stigma. Eva’s fear of additional weight gain was not actively addressed, in part due to lack of focus on active treatment of body image concerns in FBT, and in part due to Eva’s tendency to avoid discussing topics that contributed to anxiety, specifically feelings about her shape and weight. However, Eva’s developmental maturity upon second presentation empowered her to make decisions about her care, including how to involve her parents.

She ultimately was able to achieve full remission from BED over the course of 6 months, reporting significant reduction in overvaluation of her shape and weight, a sense of self-efficacy around eating flexibly, finding forms of physical movement that she enjoyed and were not compulsive, and maintaining weight around the 90th percentile. She preferred not to engage in many common body image-related skills, instead prioritizing other values (e.g., relationships and education), a focus of CBT-E. Other modalities were also drawn from to further strengthen her connection with these aspects of identity (Mindfulness-Based Self-Compassion and Acceptance and Commitment Therapy). Thus, she did not participate in many behavioral activities included within CBT-E, an adaptation that was agreed upon to empower Eva to make informed decisions about her treatment goals.

**Table 1:** Experiences, challenges, and adaptations made during ED treatment to respond to weight stigma

	Megan	Emily	Eva
Etiology	• Weight-based teasing; bullying • Internalized weight bias contributed to restriction, purging (self-induced vomiting, laxative misuse), binge eating, chewing and spitting	• Preference for foods parents considered “unhealthy” • Significant physical activity despite low weight	• Weight-based teasing; comments about body size by caregivers and peers • Internalized weight bias contributing to restriction and exercise
Diagnosis and Assessment	• Delayed ED diagnosis related to size/weight bias within assessment practices (i.e., SCOFF, PCP visit), despite high weight suppression	• Parents and doctors unconcerned about chronic poor nourishment and low weight, delaying diagnosis of ARFID • Athlete status contributed to reduced concern about low weight	• Not observed
Medical Care and ED treatment	• Delayed referral to specialized ED care • Early discharge from residential ED treatment due to insurance denying coverage related to weight-bias embedded within policies • Continued encouragement to “eat healthy” and lose weight before and after treatment	• Disagreement between caregivers and medical providers regarding increasing caloric intake and weight goal • Anxiety about transgressing family’s food-related values and becoming “unhealthy”	• Terminated care at 55th BMI%ile despite historically tracking at 89th BMI%ile • 1.5 years following completion of FBT, relapse into restriction and exercise following weight gain
Family and Peer Response	• When expressed distress about weight, shape, and size, family encouraged exercise and healthy eating as means to change body composition	• Caregivers equating health and morality with being “thin and fit”	• Culturally normative terms of endearment were experienced by patient as distressing • Focus on eating “healthy foods” • Comments about not eating “too much” when trying to prevent binge eating • Parent attempted to restrict access to certain foods to prevent weight gain and distress related to binge eating
Clinical Challenges	• In-session weighing distressing • Reaching TGW “shocking” to patient, who reported feeling that she was not “sick” enough • Ongoing body dissatisfaction challenging to address given bullying and stigma • Continued avoidance of social activities involving movement (e.g., sports)	• Patient and caregivers experienced anxiety related to weight gain in treatment • Patient reported weight gain conflicted with family's’ identified values • Difficulty understanding “flexible eating” in the context of ARFID treatment	• FBT was concluded while patient remained weight-suppressed, given patient demonstrated little distress and eating was normalized • Patient unwilling to know weight or participate in body image exposures • Interaction of stigmatizing experiences, cultural identity, experiences of minority stress, trauma, internalized weight bias, and disordered eating
Modifications to address weight stigma during treatment	• Blind weighing • Shift from FBT to CBT-E due to ongoing and persistent body dissatisfaction • Addition of psychoeducation on body diversity and weight stigma	• Psychoeducation on weight stigma to address caregiver concern for "unhealthy” foods being accepted for meals	• Blind weighing • Avoided body image-related exposures and focused on values over changing body image • Incorporating weight stigma in CBT-E formulation • Psychoeducation about weight stigma and HAES • Self-compassion to mitigate internalized weight stigma • Trauma-informed formulation of ED and interventions
Recovery	• Negative comments by peers and adults about food choices • Continued messages from medical providers about weight and diet	• Not observed	• Confusion around TGW and not enforcing weight gain beyond mBMI, despite a higher historical weight • Reluctance to gain further weight upon first admission

Note: ARFID = avoidant restrictive food intake disorder; BMI = body mass index; CBT-E = Enhanced Cognitive Behavior Therapy; ED = eating disorder; FBT = Family-Based Treatment; HAES = Health at Every Size; PCP = primary care physician; SCOFF = Sick-Control-One Stone-Fat-Food Assessment for Eating Disorders; TGW = treatment goal weight

## Discussion

Weight stigma permeates our culture and systems of care (Bristow et al., 2020; Hardy, 2022; Kornfield et al., 2015), rather than representing the fault of any individual healthcare provider or family member, all of whom typically want the best for their patient or child. The current manuscript presents three cases that demonstrate the iatrogenic effects of weight stigma during ED treatment and interactions with the broader healthcare system. While illustrative of three lived experiences, these patients’ stories are not outliers (Eiring et al., 2021; Harrop et al., 2021). Weight stigma can negatively impact adolescents with EDs in many domains, including etiology, detection and diagnosis, treatment access and course, and relapse. Given widespread impact (Harrop et al., 2023), we make several recommendations for treatment adaptations, advocacy, and research to reduce the harms of weight stigma.

Beyond what is known to play a role in the etiology of EDs (Schaumberg et al., 2017), growing research (Cerolini et al., 2024) illustrates that weight stigma may contribute to ED onset and should be a focus of future research on etiological models of EDs. In light of the recent rise in ED rates (Taquet et al., 2022) and consistent data suggesting that early detection of EDs corresponds with better prognosis (Le Grange et al., 2014), existing ED prevention efforts may be enhanced by including interventions that target weight stigma. Weight stigma also contributes to issues with ED detection and diagnosis, as observed in each of the cases discussed (Lebow et al., 2015; Sim et al., 2013). Weight-centric approaches to determining ED acuity should be de-prioritized, along with the overemphasis on BMI as a measure of health, to mitigate the negative impacts of weight stigma on diagnosis, assessment, referral to care, and insurance coverage. Instead, ED severity might be better assessed via rate and amount of weight loss (Garber et al., 2019), weight suppression or distance away from one’s historical growth curve (Jhe et al., 2022; Singh et al., 2021), and behavioral markers (Dang et al., 2023; Forbush et al., 2018). Given its positive correlation with ED severity and its prediction of ED onset (Lowe et al., 2018), weight suppression may be an especially pertinent variable in assessment and diagnosis in the context of weight stigma and mixed beliefs about weight loss for health. However, more conclusive research on ED screening and assessment strategies is needed, including among non-ED-specialists across interdisciplinary fields.

Issues of weight stigma can impact ED treatment even when patients are properly referred and gain access to evidence-based care. Although manualized ED treatments explicitly note that weight should not be the only prognostic indicator of health or recovery, and that reduction in ED thoughts and behaviors are also critically important, target goal weight ranges are often established by clinicians or dietitians at the start of treatment. With no consensus in manualized ED treatments (Dalle Grave & Calugi, 2020; Lock & Grange, 2015) on how to set target goal weights for adolescents in larger bodies (Jhe et al., 2022), a provider not informed about weight stigma may prescribe a goal weight at the 50th percentile for median BMI, despite the patient historically tracking at or above the 75th BMI percentile, and thus reducing the likelihood of recovery given recovery is contingent on returning to one’s appropriate weight range (Harrop et al., 2023; Peebles & Sieke, 2019). Developing guidelines for a personalized approach that rejects invalid BMI-based categories and weight-based population norms would enhance care collaboration, likelihood of recovery, and potentially quell anxiety experienced by adolescents and caregivers who may have experienced weight-based teasing and weight-loss counseling by righting misperceptions around health and body size.

Beyond setting goal weights, evidence-based treatments do not guide clinicians on how to incorporate weight stigma into case conceptualization or how to uphold evidence-based principles while avoiding harm. While providing care to the adolescents and their families in the case examples, clinicians exercised flexibility within fidelity by learning from past clinical experiences, and frequently consulting with other experts on the team. For example, weight stigma was incorporated into case conceptualization and there were adjuncts (e.g. using self-compassion, psychoeducation), adaptations (e.g., modified exposures for body image, providing blind weights), and omissions (e.g., exposure for body image) from established treatment manuals throughout ED treatment.

In adapting therapy to reduce weight stigma, treatment manuals could be modularized to address issues of weight stigma and draw from models of trauma-informed care or minority stress approaches. For instance, weight-shaped teasing or bullying may be combatted through a trauma-informed lens that emphasizes positive adult connections, universal norms that provide psychoeducation on weight stigma, and the unique neurodevelopment of adolescents (e.g., Bruce Perry’s three-step model: regulate-relate-reason; Nixon & Linkie, 2023; Perry, 2014). Relapse prevention could also include an explicit focus on responding to weight stigma during and after treatment. In particular, youth in larger bodies during and after weight restoration remain vulnerable to ongoing experiences of stigma or iatrogenic weight counseling, which also increases risk for relapse. Research testing the efficacy of these proposed adaptations is needed to inform clinical decision-making and promote consistency in care.

Despite these challenges, the landscape is far from bleak. Recent literature and shared clinical experience suggest that many in the field recognize the need to actively address weight stigma within general and ED-related care (Dimitropoulos et al., 2019; Kimber et al., 2019; Kramer, 2023; McEntee et al., 2023; Tylka et al., 2014). Future research should include individuals of all body sizes in treatment studies (Strand et al., 2020) and assess barriers and facilitators to long-term treatment outcomes. Longitudinal research would provide consensus on how to set goal weights and which interventions may cause more harm among individuals in larger bodies (Jhe et al., 2022; Lin et al., 2023). Lastly, assessing weight stigma experienced throughout ED treatment would enable continued evidence-based adaptations to care.

It is also essential that healthcare providers receive education that dispels myths about associations between body size, health, and EDs (see Hill et al., 2021; Moore et al., 2022; Pearl, 2018). Research to date suggests that interventions that offer sensitivity training and invoke empathy, educate about weight bias and address social norms, dispel myths about weight loss and health outcomes, and challenge individual responsibility for weight in favor of environmental and biological causes show promising outcomes, albeit studies on this lack long-term findings (Alberga et al., 2016a, 2016b; Moore et al., 2022; Pearl, 2018). Additional literature suggests that population-based approaches (e.g. anti-discrimination laws and changes in media coverage (e.g. increased body diversity and curbing stigmatizing content related to weight)) would reduce weight stigma through a downstream effect and bolster the impact of interventions designed to reduce weight stigma (Alberga et al., 2016a, 2016b; Pearl, 2018). Several international medical organizations have signed a pledge aiming to eliminate weight bias and stigma during care (Rubino et al., 2020), which could also signal a commitment to reduce weight stigma and influence other institutions. Specific to ED treatment, many programs use a weight neutral approach and focus on historic growth to address concern for weight stigma in conjunction with established evidence-based approaches (Bacon & Aphramor, 2011; Peebles & Sieke, 2019; Tylka et al., 2014). Formal assessment of this practice and long-term impacts would enhance extant evidence in our field.

## Limitations

The novelty of weight stigma research and its wide-ranging impact on individuals throughout ED treatment prohibits conclusive and exhaustive recommendations on how to reduce weight stigma during treatment. All adolescents were receiving care in the same treatment clinic employing a weight-neutral approach. These case examples do not depict experiences of all adolescents with diverse identities or their experience of ED treatment in different settings. Further, we did not seek informed consent from patients, in part due to concerns for increasing healthcare trauma. While the study was not human subjects research, and case descriptions were deidentified to protect patients and families, we note that obtaining informed consent from patients is generally best ethical practice.

## Conclusions

While there is growing consensus on considering a client's diverse identities and experiences of discrimination, oppression, and privilege within case conceptualization and treatment planning, these dialogues remain fairly limited in their consideration of body size (Vargas et al., 2020). As evidenced by the three cases and emerging research, weight stigma can have iatrogenic effects during existing evidence-based ED treatments (Harrop et al., 2023; Lebow et al., 2019; Sim et al., 2013). Opportunities to prevent EDs are missed if we do not consider and intervene on weight stigma. Further, providers may do harm by considering weight alone in assessing ED severity and diagnosis (Golden & Walsh, 2024; Hebebrand et al., 2024) or not tailoring care to fit patient needs. Treatment providers make treatment adaptations in the service of helping those with whom they work, and there are unfortunate limits in the healthcare system that preclude treatment access, dissemination of interventions, and inclusive research that hinder growth, despite good intent. Fortunately, there is evidence of positive movement in the field and treatment adaptations, more inclusive research, wider-reaching training, and advocacy efforts are needed to reduce weight stigma in ED care and more generally.

## Supplementary Information

Below is the link to the electronic supplementary material.

Supplementary file1 (DOCX 17 KB)

## Footnotes

1 Median BMI is derived from CDC data and based on the 50th percentile weight for age and sex (Society for Adolescent Health and Medicine, 2022).

2 The SCOFF (Morgan et al., 2000) was used and Megan did not endorse enough items (out of 5) to meet the cut-off score.
