# Supplementary material for: Weight Stigma in the Development, Maintenance, and Treatment of Eating Disorders: A Case Series Informing Implications for Research and Practice
Source: Res Child Adolesc Psychopathol. 2024 Nov 1;53(5):747–60. doi: 10.1007/s10802-024-01260-3 (PMC12043962; doi:10.1007/s10802-024-01260-3)
Supplement: Supplementary file 1 — Supplementary file1 (DOCX 17 KB) [file 10802_2024_1260_MOESM1_ESM.docx]

Supplement 1: Overview of Evidence-Based ED Treatments Utilized in Case Series

**Enhanced Cognitive Behavior Therapy** (CBT-E; Fairburn, 2008) is a transdiagnostic outpatient therapy first developed for adults with EDs and later manualized for youth (Dalle-Grave & Calugi, 2020). CBT-E is divided into four stages. The first stage focuses on treatment engagement and motivation, psychoeducation about EDs, and the collaborative creation of a formulation of factors that appear to be maintaining the ED. Individuals then work on normalizing eating and restoring weight if needed by recording nutrition and ED-related thoughts, feelings, and behaviors. The second stage provides the opportunity for therapist and patient to "take stock” of progress and identify barriers to change. In stage three, other maintaining ED mechanisms are addressed, including dietary restraint and avoided foods, body image concerns, and moods and events that precipitate ED symptoms. Finally, stage four focuses on short- and long-term relapse prevention; additional modules related to perfectionism, interpersonal difficulties, low self-esteem, and depressive symptoms are also available.

**Family Based Treatment** (FBT; Lock & Le Grange, 2015) is an outpatient treatment first developed for youth with anorexia nervosa, and later extended to other EDs. FBT engages caregivers and other family members to help their child overcome ED. Caregivers temporarily take charge of nutrition, supporting their child to restore weight and reduce eating disorder behaviors. The clinician acts as a consultant, leveraging their expertise in EDs to support caregiver self-efficacy. Phase I focuses on renourishment and reduction in ED behaviors (e.g., purging) with caregivers generally having full responsibility for nutrition and supervision. Phase II focuses on a return to independent eating, and Phase III focuses on a return to typical adolescent development and relapse prevention.

**Cognitive Behavioral Therapy- Avoidant Restrictive Food Intake Disorder**

(CBT-AR; Thomas & Eddy, 2018) is an outpatient treatment specifically for ARFID. It combines principles of FBT (e.g., caregiver nutritional support) and CBT (e.g., exposures, cognitive restructuring). Stage one focuses on psychoeducation, the collaborative creation of a formulation of factors that appear to be maintaining the ARFID, and small changes toward regular eating and nutritional variety. In stage two, the therapist and patient set goals. Stage three involves exposures focused on food variety, feared aversive consequences of eating (e.g., choking, vomiting), and/or other treatment targets. Stage four focuses on relapse prevention.

Of note, all treatments involve open weights, where patients see their weight each session.
